# The First Year Experience of Newborn Screening for Pompe Disease in California

**DOI:** 10.3390/ijns6010009

**Published:** 2020-02-07

**Authors:** Hao Tang, Lisa Feuchtbaum, Stanley Sciortino, Jamie Matteson, Deepika Mathur, Tracey Bishop, Richard S. Olney

**Affiliations:** Genetic Disease Screening Program, California Department of Public Health, 850 Marina Bay Parkway, MS 8200, USA; lisa.feuchtbaum@cdph.ca.gov (L.F.); stanley.sciortino@cdph.ca.gov (S.S.); jamie.matteson@cdph.ca.gov (J.M.); deepika.mathur@cdph.ca.gov (D.M.); tracey.bishop@cdph.ca.gov (T.B.); richard.olney@cdph.ca.gov (R.S.O.)

**Keywords:** Pompe disease, newborn screening, California

## Abstract

The California Department of Public Health started universal newborn screening for Pompe disease in August 2018 with a two-tier process including: (1) acid alpha-glucosidase (GAA) enzyme activity assay followed by, (2) *GAA* gene sequencing analysis. This study examines results from the first year of screening in a large and diverse screening population. With 453,152 screened newborns, the birth prevalence and GAA enzyme activity associated with various types of Pompe disease classifications are described. The frequency of *GAA* gene mutations and allele variants are reported. Of 88 screen positives, 18 newborns were resolved as Pompe disease, including 2 classic infantile-onset and 16 suspected late-onset form. The c.-32-13T>G variant was the most common pathogenic mutation reported. African American and Asian/Pacific Islander newborns had higher allele frequencies for both pathogenic and pseudodeficiency variants. After the first year of Pompe disease screening in California, the disease distribution in the population is now better understood. With the ongoing long-term follow-up system currently in place, our understanding of the complex genotype-phenotype relationships will become more evident in the future, and this should help us better understand the clinical significance of identified cases.

## 1. Introduction

Pompe disease is a sometimes-fatal inherited lysosomal storage disorder caused by the abnormal accumulation of glycogen in cells, which can result in progressive dysfunction of the heart and other muscles. Also known as glycogen storage disease type II, Pompe disease is caused by a deficiency of the acid alpha-glucosidase (GAA) enzyme that breaks down a type of complex sugar, lysosomal glycogen. The birth prevalence of Pompe disease has been estimated to be 1 in 40,000 [1,2], or 25 per 1 million births, although studies from Israel, Taiwan and some parts of the United States reported higher prevalence rates [3,4,5].

The severity, age of onset, and rate of progression of Pompe disease vary among individuals, who have been generally categorized into three types. The classic infantile-onset Pompe disease (IOPD) shows symptoms within a few months of birth, characterized by fatal cardiomyopathy if untreated. The non-classic infantile-onset form begins before age one, typically with no heart complications. The late-onset Pompe disease (LOPD) appears later in childhood, adolescence, or adulthood [6,7,8]. The variance in phenotypes has been linked to different *GAA* gene variants, which are the cause for GAA enzyme deficiency. Certain pathogenic variants on both *GAA* alleles severely reduce GAA activity and usually lead to IOPD. On the other hand, some variants of the *GAA* gene exhibit low levels of GAA activity, leading to more moderate forms of Pompe disease [1,9]. To date, enzyme replacement therapy (ERT) has been the only direct medical treatment for all forms of Pompe disease by reducing GAA deficiency. Treatment beginning as soon as the disease is detected, or as early as possible, can generate the most benefit for patients [10,11,12,13,14].

The clinical work-up of Pompe disease usually involves measuring GAA enzyme activity and molecular analysis to confirm the diagnosis [5,15]. Recent studies have shown that a tandem mass spectrometry (MS/MS)-based GAA enzyme activity assay could be a functional laboratory method for Pompe disease detection [16,17]. The option of multiplex testing for Pompe disease, along with other MS/MS disorders using the same dried blood spot (DBS), helped promote Pompe disease as a viable disorder to add to newborn screening panels [18,19]. The first newborn screening program for Pompe disease was implemented in Taiwan as early as 2005 [20]. Since then, several other countries and U.S. states have conducted pilot screening studies with promising results [5,21,22,23]. Subsequently, an external condition review workgroup commissioned by the Health Resources and Services Administration examined the evidence for including Pompe disease on the federal Recommended Uniform Screening Panel (RUSP) in 2013 [24]. In 2013, the Advisory Committee on Heritable Disorders in Newborns and Children voted to recommend that the United States Secretary of Health and Human Services add the disorder to the RUSP, which occurred in March 2015 [25]. As of November 2019, 22 states are screening for Pompe disease [26]. 

The addition of Pompe disease to California’s Newborn Screening (NBS) panel followed passage of SB 1095 in the California legislature in 2016 that amended the Health and Safety Code [27,28]. This required the Genetic Disease Screening Program (GDSP) of the California Department of Public Health to add Pompe disease in order to be compliant with the RUSP, and this process has been described in more detail by Bronstein et al. [29]. On August 29, 2018, California began universal screening for Pompe disease. 

This paper reports the findings from the first year of population-based Pompe disease screening. We describe our screening and follow-up algorithm as well as epidemiological and clinical outcomes of screening, including disease and variant classification and other characteristics of the Pompe cases identified to date.

## 2. Materials and Methods 

**Figure 1:**
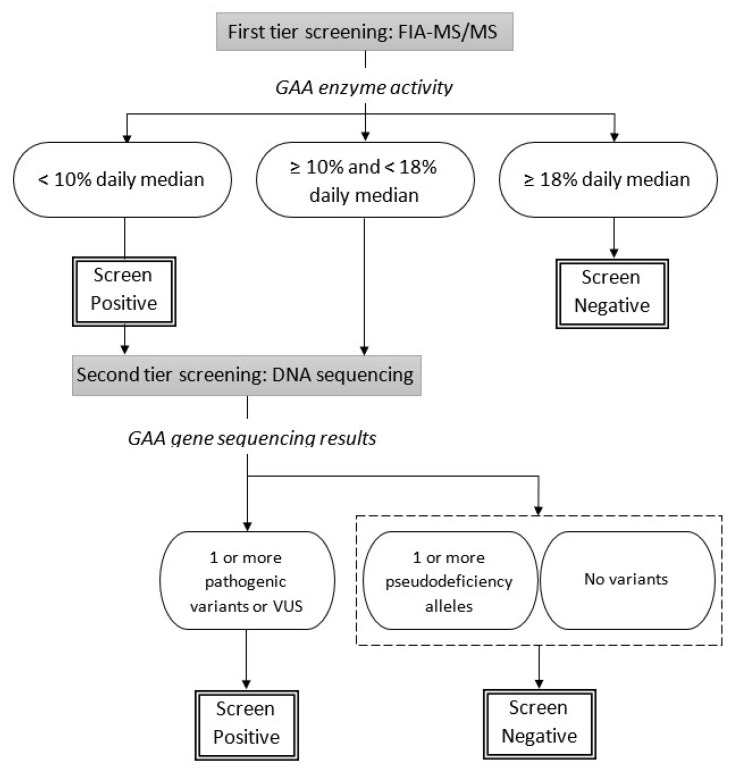
California Pompe disease newborn screening algorithm.

In California, Pompe disease screening is a two-tier process as shown in Figure 1. DBSs are analyzed using flow injection analysis-tandem mass spectrometry (FIA-MS/MS) to measure GAA enzyme activity. Specimens whose GAA enzyme activity levels are below 18% of the daily median are separated into two groups. Those with very low levels, below 10% of the daily median, are immediately called out as screen positive and sent for *GAA* gene sequencing and clinical follow-up, while those with intermediate GAA enzyme levels, between 10% and 18% of the daily median await *GAA* gene sequencing results before the final interpretation is made. The specimens with intermediate GAA enzyme levels are only referred for clinical follow-up if at least one pathogenic variant, likely pathogenic variant or variant of uncertain significance (VUS) is found. 

Clinical follow-up is conducted in one of fifteen metabolic specialty care centers across California. The specialty care centers provide genetic counseling, confirmatory testing, diagnosis, and long-term clinical care when appropriate.

All testing results (biochemical and DNA sequencing), along with demographic information and follow-up reports (short-term follow-up to diagnosis and long-term follow-up for five years), associated with the referred newborns are entered and stored in GDSP’s web-based Screening Information System (SIS), including a newborn screening registry that houses all clinically confirmed Pompe disease cases. The categories of the California Pompe disease case resolutions include: (1) classic infantile-onset Pompe disease (with cardiac involvement), (2) non-classic infantile-onset Pompe disease (without cardiac involvement), (3) late-onset Pompe disease, and (4) not-otherwise-specified Pompe disease. After referral, metabolic specialists make the diagnostic decision following established case definitions [30] and general guidelines (Table 1). Newborns who are carriers or who only have pseudodeficiency alleles are also recorded in the registry for reference, but these newborns are not referred for additional clinical follow-up. Variant classification of pathogenic, likely pathogenic, uncertain significance, and pseudodeficiency allele are based on established guidelines with published *GAA* mutations [31,32,33]. For some of the analyses, we combined late-onset and not-otherwise-specified Pompe disease cases into a “suspected late-onset” category due to the similarities of their diagnostic characteristics (both had no symptoms and had similar GAA levels and variants).

We used California newborn screening data collected from 29 August 2018 through 31 August 2019. We described neonatal characteristics of all screen-positives by disease category. Demographic characteristics included newborns’ sex (female, male), nursery type (Neonatal Intensive Care Unit (NICU), non-NICU), and maturity at birth (premature/<37 weeks, term/≥37 weeks). GAA enzyme activity was measured as µmol/L per hour, and the distribution of its percentage of the daily median was examined by Pompe disease categories using a box and whisker plot. We tabulated variant classification distribution across race/ethnicity groups. Race/ethnicity of each newborn was recorded as a multiple-choice check box on the GDSP Test Request Form (TRF). Single ethnic choices on the TRF were recoded to African American, Asian/Pacific Islander (API), Hispanic, non-Hispanic (NH) White, and Other. If multiple categories were reported for a newborn, we used a hierarchy to recode race/ethnicity to a single group following the order of (1) African American, (2) Hispanic, (3) API, (4) NH White, and (5) Other. Native Americans were included in the ‘other’ category. Variant classification information was reported for all diagnosed cases. Case notes and follow-up reports were abstracted and reviewed for the two classic IOPD patients.

All analyses were performed with SAS/STAT software version 9.4 of the SAS system for Windows (SAS Institute, Cary, NC, USA).

## 3. Results

### 3.1. Birth Prevalence 

During the study period, 453,152 newborns received genetic disease screening from GDSP. Based on the GAA enzyme activity cutoff (percentage of daily median <18%), 88 newborns were screen positive for Pompe disease and received *GAA* gene sequencing to analyze mutations. Among those referred, two were diagnosed with classic IOPD, and 16 had case resolution of LOPD including 11 late-onset and five not-otherwise-specified Pompe disease, indicating an overall birth prevalence of 1 in 25,200. As of the time of this reporting, we have yet to observe a non-classic IOPD case.

Table 2 shows selected characteristics of 88 Pompe disease screen positives that have a case resolution. Male infants were more likely to be called out as screen positive. Nearly 40% of Pompe disease positive infants were in the NICU when the blood specimens were drawn, while in general, around 10% of infants were in the NICU statewide. Both classic IOPD newborns were in the NICU, and only two suspected LOPD infants needed intensive care. Interestingly, 11 out of 20 pseudodeficiency newborns and 14 of 16 false positive (no mutations found) newborns were in NICUs, suggesting other neonatal factors might play a role in reducing GAA enzyme activity in the absence of a pathogenic *GAA* gene variant. For example, eight out of 20 pseudodeficiency newborns were born prematurely.

### 3.2. GAA Activities and Pompe Disease Diagnosis 

**Figure 2:**
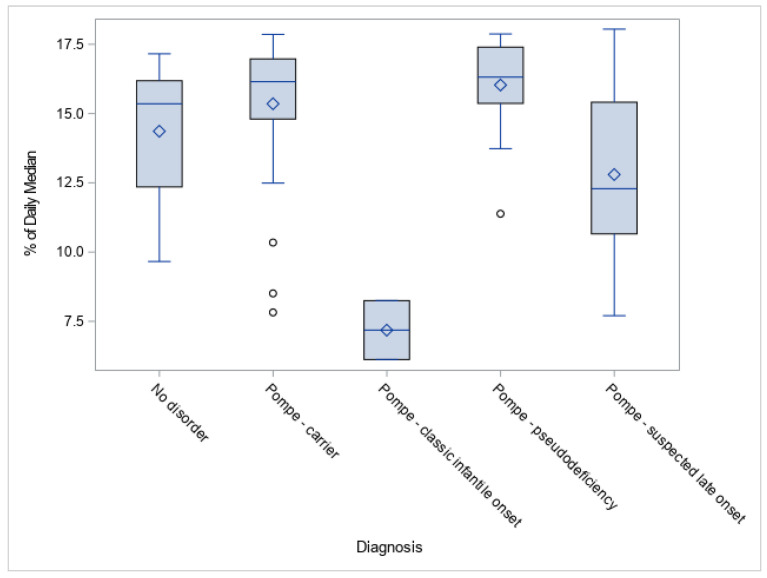
Acid alpha-glucosidase (GAA) activities (% of daily means) by resolution for positive Pompe disease screening.

A potential link between GAA activities and forms of Pompe disease was observed. GAA values for the two patients diagnosed as classic IOPD had GAA activity significantly lower than LOPD cases and other non-disease categories (Figure 2). This observation was expected based on the pathogenesis of Pompe disease. Suspected LOPD newborns had lower GAA activity compared to carrier, pseudodeficiency, and false positive categories, although with a wide range.

### 3.3. GAA Gene Mutations and Allelic Frequency

As shown in Table 3, a total of 120 *GAA* gene variants were reported among the screen-positive cases, including 52 (43.3%) pathogenic variants, 52 (43.3%) pseudodeficiency alleles, and 16 (13.3%) VUS. The c.-32-13T>G variant was the most common pathogenic mutation (34.6% of all pathogenic variants) and was present in 10 of the 16 suspected LOPD cases, followed by c.[752C>T;761C>T]. A homozygous c.1799G>A variant was found in one of the IOPD patients; the c.1979G>A variant and the c.1754+1_1754+12delinsCCA variant were found in the other. The c.[1726G>A;2065G>A] variant was the predominant pseudodeficiency allele (80.8% of all pseudodeficiency variants).

The overall pathogenic allele frequency was 115 per million (or 1 in 8700) in California’s NBS population. Asian and Pacific Islander (API) and African American newborns had relatively higher frequencies (216/1,000,000 and 161/1,000,000, respectively). The overall pseudodeficiency allele frequency was also 115 per million, with API having a significantly higher rate of 432 per million (1 in 2300). Relatively higher frequencies of VUS were found in API and African American as well (Table 4).

### 3.4. Diagnosed Cases and Case Study of IOPD Patients

Of the 18 infants diagnosed with Pompe disease (IOPD and suspected LOPD), 12 had either a homozygous pathogenic variant or a pair of distinctive pathogenic/likely pathogenic variants (Table 5). The other six had at least one VUS, indicating a less conclusive diagnosis. Three of the 18 diagnosed cases also had a pseudodeficiency allele.

We examined the testing results and follow-up reports on the two IOPD cases.

Case 1: This is an infant with homozygous pathogenic variant c.1799G>A, a known pathogenic mutation linked to IOPD [33,34]. The GAA confirmatory test showed “markedly reduced” enzyme activity. Further confirmatory testing showed urine glucose tetrasaccharide quantitation (Hex4) was elevated. Hypertrophic cardiomyopathy and arrhythmia were noted on the service report provided by the metabolic specialty care center clinical staff. ERT was started at two months of age.

Case 2: This is an infant with two heterozygous pathogenic variants. The c.1979G>A variant has been associated with both IOPD and LOPD [35,36]; and the c.1754+1_1754+12delinsCCA variant has no reported link to Pompe disease but was deemed as disease-causing in general [37]. Confirmatory tests found reduced GAA enzyme activity and mildly elevated Hex4. Abnormal echocardiogram and electrocardiogram results, as well as hypertrophic cardiomyopathy, were reported at the time of diagnosis. We confirmed that ERT was started but the exact starting age was unclear.

## 4. Discussion

The present study is one of the first reports on statewide Pompe disease screening outcomes after its placement on the RUSP, especially with a relatively large population base. California GDSP screened almost half a million babies in its first year (2018–2019) and of those referred, indicated a birth prevalence of 1 in 25,200 (IOPD and LOPD combined), which is within the range of previously reported prevalence. However, due to the rare occurrence of the disorder in the general population, only a small number of cases were reported, thus limiting the accuracy of birth prevalence calculation. With only two cases of IOPD, the birth prevalence in California (approximately 1 in 250,000) was lower than in other regions (1 in 138,000 in the Netherlands [38], 1 in 50,000 Taiwan [4,6], or 1 in 4500 in Maroon population of French Guiana [39]). However, the prevalence of potential LOPD (approximately 1 in 37,500) seems to be higher than the previously reported prevalence among the Dutch population (1 in 57,000) [38], but lower than that of Taiwan (approximately 1 in 25,000) [6]. Based on the birth prevalence of diagnosed Pompe disease cases, the calculated carrier frequency using the Hardy-Weinberg principle indicates more than five thousand carriers in our screened population. The number of carriers (34) identified from NBS was significantly fewer than that estimate because the cutoff of GAA activity in NBS aims at identifying Pompe disease cases, which have significantly lower GAA enzyme activity than that of carriers. 

Six of the 16 suspected LOPD cases had at least one VUS, and since none of them have exhibited symptoms, some of their diagnoses could be changed to carrier, pseudodeficiency or no disorder based on the future clinical follow-up results. The inherent uncertainty of VUS results leads clinicians to cautiously diagnose a late-onset disorder, but affected children and their families might endure years of anxiety due to the unknown pathogenicity and consequence of the molecular findings [40]. Except when symptoms are clearly identified and a diagnosis has been made by a specialist, our observations are preliminary and incomplete given the short follow-up period of this study.

The diagnosis of Pompe disease identified by NBS is largely based on the results from molecular analysis along with supportive confirmatory testing, especially for patients who have not exhibited any symptoms. The high occurrence of the pathogenic c.-32-13T>G variant in our screen positive samples (40.4% of all pathogenic variants) echoed findings from literature, which reported an allelic frequency from 40% to 70% [41]. For newly screened rare disorders with late-onset phenotypes like Pompe disease, one of the greatest challenges for screening is the VUS category in which cases have an unknown pathogenic molecular profile. Some VUS may eventually be recognized as pathogenic, but barriers to receiving a thorough clinical work-up or ongoing clinical follow-up (such as factors associated with access to care), could play a role in obtaining a more definitive diagnosis later. With a more developed global registry and variant database [32,34] future screening could yield more predictive results.

California has a vastly diverse population. In our study, Pompe disease-positive newborns with Asian and Pacific Islander (API) ancestry had a high occurrence of pseudodeficiency alleles, especially the c.[1726G>A;2065G>A] variant, which represents 80% of all the pseudodeficiency mutations. This finding confirmed the results from other studies with Asian populations [22,42,43]. Unlike these other studies, we did not find any Pompe disease cases (IOPD or LOPD) among nearly 70,000 API newborns, and we only found one c.1935C>A (linked to c.[1726G>A;2065G>A]), which was identified as the most common pathogenic *GAA* variants among Asian countries. African American newborns had a birth prevalence of 54 per million (or 1 in 18,700), which was the highest among all groups. This result may be indicative of a potentially high Pompe disease birth prevalence among African Americans, but more data are needed to be conclusive. Previous research identified c.2560C>T as the most common *GAA* variant among African Americans [34,44]. We did not have a large enough sample size (*n* = 7) of African American infants who had variants to confirm the finding. The only c.2560C>T variant, however, was indeed detected in an African American sample.

In most of the study period (before 21 August 2019), every newborn with GAA activity < 18% of the daily median was flagged as an urgent call-out by the laboratory before the results of *GAA* gene sequencing was available. About six months after the Pompe disease newborn screening began, NBS received communications from clinical specialists about the follow-up burden for both patients and providers due to the large number of patients being referred; many of them were either pseudodeficiency or no mutation based on the sequencing findings. Although previous research showed that MS/MS analysis of GAA activity could separate pseudodeficiency and Pompe disease cases [45], our screening test results still showed some overlap in GAA activity for these two groups. GDSP evaluated the available data and modified the protocol in August 2019 to flag only the cases with GAA activity less than 10% of the daily median for urgent call-out (the two IOPD cases identified through the program were well-below this threshold). Newborns with GAA activities between 10% and 18% of the daily median are only referred if the molecular results show pathogenic, likely pathogenic, or VUS mutations. In other words, we wait so that screen-positive newborns with homozygous or heterozygous pseudodeficiency alleles or no mutations are not referred to the specialty care centers for further follow-up. This serves as a good example of how synergy between providers and the newborn screening program minimized the unnecessary referrals and improved screening performance. If we later find infants who have GAA activities between 10% and 18% of the daily median diagnosed with IOPD, we will consider adjusting the cutoff again for urgent call-outs.

More than four years after Pompe disease was added to the RUSP, the adoption and implementation of newborn screening at the state level has been at a moderate pace. In the first year of Pompe disease newborn screening in California, we have gained a better understanding of the disease distribution at the population level, and most importantly, now have experience and evidence to support effective screening. With a robust long-term follow-up component, GDSP values the necessity of monitoring all potential cases, including those with a VUS [46]. The growing knowledge from long-term follow-up will further improve our understanding of the clinical significance of these cases, especially when case management algorithms are still undeveloped for asymptomatic patients [47].

While the two newborns with IOPD were identified while in the NICU, almost all of the newborns with LOPD were identified in the regular nursery. These newborns were asymptomatic and unlikely to be identified as at risk for Pompe disease except by screening. Now that treatment is warranted before symptoms develop, the value of population-based screening is clear: to identify the youngest candidates for treatments that can reduce life-long disability [48].

## Figures and Tables

**Table 1:** California newborn screening Pompe disease diagnosis guideline.

Diagnosis	Mutation Status	Symptoms	Long-Term Follow-Up
Pompe–classic infant onset (with cardiac involvement) *	Pathogenic/likely pathogenic/VUS alleles ** ≥ 2	Yes, with positive cardiac involvement	Yes
Pompe–non-classic infant onset (without cardiac involvement) *	Pathogenic/likely pathogenic/VUS alleles ** ≥ 2	Yes, without positive cardiac involvement	Yes
Pompe–late onset Pompe disease *	Pathogenic/likely pathogenic/VUS alleles ** ≥ 2	No	Yes
Pompe–not otherwise specified *	Pathogenic/likely pathogenic/VUS alleles ** ≥ 2	No	Yes
Pompe–carrier	Pathogenic/likely pathogenic/VUS alleles = 1	No	No
Pompe–pseudodeficiency	Pseudodeficiency alleles	No	No
No disorder	No mutation found	No	No

* Regardless of the presence of pseudodeficiency allele, ** Any combination of pathogenic-pathogenic, pathogenic-likely pathogenic, pathogenic-VUS, likely pathogenic-VUS, and VUS-VUS.

**Table 2:** California Pompe disease screening results (among screen positives) by neonatal factors.

	Classic Infantile-Onset	Suspected Late-Onset	Carrier	Pseudo-Deficiency	No Disorder	Overall
**Sex**						
Female	1	5	14	11	6	37
Male	1	11	20	9	10	51
**Nursery**						
NICU	2	1	4	11	14	32
Non-NICU	0	15	30	9	2	56
**Maturity**						
Premature	1	2	4	8	2	17
Full term	1	14	30	12	14	71
**Total**	2	16	34	20	16	88
**Birth prevalence**	5/1,000,000	36/1,000,000	75/1,000,000	45/1,000,000		
	(1 in 226,600)	(1 in 28,300)	(1 in 13,300)	(1 in 22,700)		

**Table 3:** Pompe disease variants identified by California newborn screening.

Mutation Name	Count
Pathogenic variant	
c.-32-13T>G	18
c.[752C>T;761C>T] *	8
c.2238G>C, c.1099T>C, c.1799G>A, c.1437+1G>A, c.1548G>A, c.1579delA, c.1754+1_1754+12delinsCCA, c.1856G>A, c.1933G>A, c.1935C>A, c.1979G>A, c.2297A>C *, c.2408_2426del19, c.2560C>T, c.2646+2T>A, c.29delA, c.511del, c.546G>A, c.546G>C, c.573C>A, c.670C>T, c.925G>A	<5
Subtotal	52
Pseudodeficiency allele	
c.[1726G>A;2065G>A]	42
c.2065G>A	5
c.271G>A	5
Subtotal	52
Variant of uncertain significance	
c.1048G>A, c.1019A>G, c.1357G>A, c.1375G>A, c.1392_1393delinsTT, c.1477C>T, c.1757C>T, c.2221G>A, c.2261C>T, c.265C>T, c.266G>A **, c.316C>T, c.546+5G>T, c.726G>A, c.868A>G	<3
Subtotal	16
Total	120

* Noted as presumably non-pathogenic in the updated Pompe variant database: http://pompevariantdatabase.nl. ** Noted as presumably non-pathogenic but pathogenic with a null allele in the updated Pompe variant database: http://pompevariantdatabase.nl.

**Table 4:** Allelic frequency by race/ethnicity.

	Pathogenic		Pseudodeficiency Allele		Uncertain Significance	
Race/Ethnicity	Count	Allele Frequency	Count	Allele Frequency	Count	Allele Frequency
African American (*n* = 37,340)	6	161/1,000,000 (1 in 6200)	4	107/1,000,000 (1 in 9300)	3	80/1,000,000 (1 in 12,500)
Asian/Pacific Islander (API, *n* = 69,510)	15	216/1,000,000 (1 in 4600)	30	432/1,000,000 (1 in 2300)	6	86/1,000,000 (1 in 11,600)
Hispanic (*n* = 214,049)	14	66/1,000,000 (1 in 15,300)	7	33/1,000,000 (1 in 30,600)	5	23/1,000,000 (1 in 42,800)
White (*n* = 115,281)	17	148/1,000,000 (1 in 6800)	11	95/1,000,000 (1 in 10,480)	2	17/1,000,000 (1 in 57,600)

**Table 5:** Mutation status of diagnosed cases identified by California newborn screening.

Diagnosis	Number of Cases	Mutation Status
Pompe—classic infantile onset	1	Pathogenic, homozygous
	1	Pathogenic & pathogenic
Pompe—suspected late onset	3	Pathogenic, homozygous
	7	Pathogenic & Pathogenic/likely pathogenic
	4	Pathogenic & VUS
	1	VUS, homozygous
	1	VUS & VUS
